# Population structure of the malaria vector *Anopheles moucheti *in the equatorial forest region of Africa

**DOI:** 10.1186/1475-2875-7-120

**Published:** 2008-07-04

**Authors:** Christophe Antonio-Nkondjio, Cyrille Ndo, Pierre Kengne, Louis Mukwaya, Parfait Awono-Ambene, Didier Fontenille, Frédéric Simard

**Affiliations:** 1 Laboratoire de Recherche sur le Paludisme, Organisation de Coordination pour la Lutte contre les Endémies en Afrique Centrale (OCEAC), P.O. Box 288, Yaoundé, Cameroon; 2 Laboratoire de lutte contre les Insectes Nuisibles, Institut de Recherche pour le Développement (IRD), UR 016, 911, avenue Agropolis, P.O. Box 64501, 34394 Montpellier cedex 5, France; 3 Faculty of Sciences, University of Yaoundé I, P.O. Box 337, Yaoundé, Cameroon; 4 Entomology Division, Uganda Virus Research Institute, P.O. Box 49, Entebbe, Uganda

## Abstract

**Background:**

*Anopheles moucheti *is a major malaria vector in forested areas of Africa. However, despite its important epidemiological role, it remains poorly known and insufficiently studied. Here, levels of genetic differentiation were estimated between different *A. moucheti *populations sampled throughout its distribution range in Central Africa.

**Methods:**

Polymorphism at ten microsatellite markers was compared in mosquitoes sampled in Cameroon, the Democratic Republic of Congo and an island on Lake Victoria in Uganda. Microsatellite data were used to estimate genetic diversity within populations, their relative long-term effective population size, and the level of genetic differentiation between them.

**Results:**

All specimens collected in Tsakalakuku (Democratic Republic of Congo) were identified as *A. m. bervoetsi *while other samples consisted of *A. m. moucheti*. Successful amplification was obtained at all microsatellite loci within all *A. m. moucheti *samples while only six loci amplified in *A. m. bervoetsi*. Allelic richness and heterozygosity were high for all populations except the island population of Uganda and *A. m. bervoetsi*. High levels of genetic differentiation were recorded between *A. m. bervoetsi *and each *A. m. moucheti *sample as well as between the island population of *A. m. moucheti *and mainland populations. Significant isolation by distance was evidenced between mainland populations.

**Conclusion:**

High levels of genetic differentiation supports complete speciation of *A. m. bervoetsi *which should henceforth be recognized as a full species and named *A. bervoetsi*. Isolation by distance is the main force driving differentiation between mainland populations of *A. m. moucheti*. Genetically and geographically isolated populations exist on Lake Victoria islands, which might serve as relevant field sites for evaluation of innovative vector control strategies.

## Background

Malaria remains one of the world's major health problems claiming at least one million deaths each year in Africa [1]. In the forested areas of equatorial Africa, where malaria transmission occurs all year long, *Anopheles moucheti *mosquitoes can sustain malaria transmission intensities as high as 100–300 infected bites per man per year in villages located at the vicinity of large rivers and slow-moving streams where its larvae develop [2-5]. However, despite playing such an important epidemiological role in malaria transmission, this group of mosquitoes remains poorly known and insufficiently studied. Data on its bionomics and genetic structure are currently lacking although such data are of paramount importance for a comprehensive implementation and monitoring of malaria vector control in Central Africa [6].

*Anopheles moucheti *is a group of three morphological forms: *A. moucheti moucheti, A. moucheti nigeriensis *and *A. moucheti bervoetsi*, which can be distinguished by minor variations in the size and distribution of pale fringe spots and pale vein spots on the wings at the adult stage, and at the larval stage by the number of branches of the saddle hair (>5 branches for *A. m. bervoetsi *and <5 branches for the two others) [7,8]. However, population genetics studies using allozyme markers revealed that these morphological variations were not segregating between the different taxonomic units that build up the *A. moucheti *group in Central Africa and were therefore of poor diagnostic value [9]. More recently, DNA sequence differences were detected in the mitochondrial gene encoding the cytochrome B (CytB) and the ribosomal DNA Internal Transcribed Spacers (ITS) 1 and 2 between specimens of *A. m. moucheti*, *A. m. nigeriensis *and *A. m. bervoetsi *and a diagnostic PCR assay was subsequently developed allowing straightforward identification of the three taxonomic units within the *A. moucheti *group [10]. This study further suggested that *A. m. moucheti *is widespread throughout the forested areas of Central Africa, whereas *A. m. nigeriensis *and *A. m. bervoetsi *were found only in their type localities in Nigeria and the Democratic Republic of Congo (DRC), respectively. Microsatellite DNA markers have been isolated from *A. moucheti *[11] and these were demonstrated to be suitable tools for population genetics studies within this group of mosquitoes [12]. Very low levels of genetic differentiation (Fst<0.0275) were detected between *A. moucheti *populations situated 65–400 km apart in Cameroon, suggesting high levels of gene flow at this geographical scale [12].

Previous findings from Cameroon [12] are expanded through the inclusion of mosquitoes sampled in DRC and Uganda, to further explore the level of genetic structuring between populations of the *A. moucheti *group and to precise the taxonomic status of *An. m. bervoetsi*. Analytical methods, based on various aspects of the data, are used to provide insights into the role and relative importance of geographic distance, demographic parameters (*eg *effective population size and demographic instability) and natural barriers to gene flow such as habitat discontinuities and speciation in shaping the observed population structure.

## Methods

### Mosquitoes sampling and collection sites

**Figure 1:**
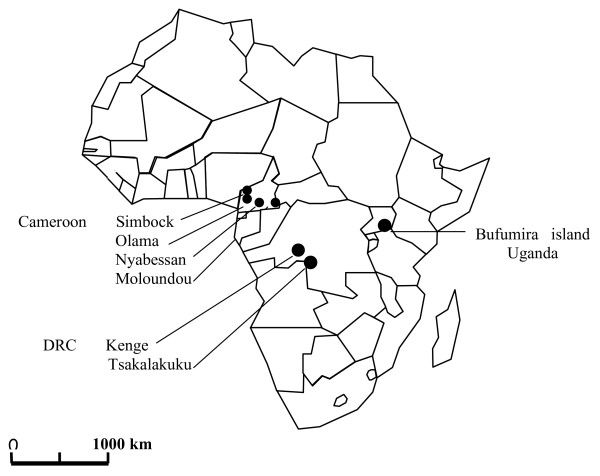
A schematic map of Africa showing sampling sites for *A. moucheti *in Cameroon, Democratic Republic of Congo (DRC) and Uganda.

The mosquito samples obtained from four villages in Cameroon that were used in this study were described in details previously [12]. Additional adult mosquitoes were collected by pyrethrum spray catches and/or bednet traps from two villages in DRC including the type locality of *A. m. bervoetsi*, Tsakalakuku (5°51'S; 17°23'E) and Kenge (5°19'S; 19°58'E); and from the island of Bufumira (0°19'S; 32°22'E) on Lake Victoria in Uganda (Figure 1). Collections were attempted in Nigeria, in and around the village of Akaka (6°27'N; 3°24'E) in the Lagos area where *A. m. nigeriensis *was originally described [7], but were unsuccessful.

Tsakalakuku is situated in the tropical wet savannas region of Africa. The area is characterized by a succession of hills covered with grass fields and valleys occupied by forest galleries along streams. The climate comprises a dry and a rainy season of six months each. All other collection sites are located within the Congo-Guinean phytogeographic zone, characterized by a typical equatorial climate with two rainy seasons extending from March to June and from September to November (total rainfall around 1,500 mm per year).

Mosquito collections were conducted from July 2003 to April 2004 in Cameroon [12], in December 2003 in DRC and in October 2004 in Uganda. *Anopheles moucheti *specimens were visually sorted from other anophelines according to morphological identification keys [7,13]. All specimens were stored individually and kept at -20°C until further analysis.

### DNA extraction and genotype scoring

Genomic DNA was extracted from wings or legs of each individual mosquito as described earlier [12]. Morphological identification was confirmed through the recently developed PCR based assay [10]. Genotypes at 10 microsatellite loci were determined for the DRC (N = 64 in Tsakalakuku and N = 11 in Kenge) and Uganda (N = 57) samples as previously described [12].

### Data analysis

Genetic diversity within samples and overall was measured at each locus by estimating allele richness *Rs*, an unbiased estimator of the number of alleles in each sample accounting for differences in sample sizes [14], and *He *[15], the unbiased expected heterozygosity under Hardy-Weinberg equilibrium (HWE), using the software FSTAT V2.9.3.2 [16]. Genotypic frequencies were tested against HWE for each locus in the pooled population and in each sample. Statistical significance was assessed by the exact probability test available in GENEPOP V3.2 [17]. Linkage disequilibrium between loci was tested by exact tests on contingency tables, also available in GENEPOP.

Genetic differentiation between populations was assessed by estimating Wright's F-statistics [18], calculated according to Weir & Cockerham [19]. Statistical significance of Fst was assessed using G-based exact tests for genotypic differentiation [20], available in GENEPOP. The correlation between genetic and geographic distances, assuming isolation by distance, was assessed by the regression of pairwise Fst estimates on the logarithm (ln) of geographic distances between sampling sites [21], and tested by the Mantel test available in GENEPOP. A Bayesian approach was further implemented to infer the number of genetic clusters (*K*) in the dataset without prior information on the sampling locations, using STRUCTURE 2.2 [22]. A model where the allele frequencies were correlated within populations was assumed (λ was set at 1, the default value). The software was run with the option of admixture, allowing for some mixed ancestry within individuals, and α was allowed to vary. Twenty independent runs were done for each value of *K *(K = 1 to 8), with a burn-in period of 100,000 iterations and 100,000 replications. The method of Evanno *et al *[23] was used to determine the most likely number of clusters. This approach uses an *ad hoc *quantity, Δ*K*, based on the second order rate of change of the likelihood function between successive values of *K*.

Because demographic instability such as recent population bottleneck and/or expansion might bias genetic differentiation estimates to a significant extent [24,25], heterozygosity tests were used to test for Mutation-Drift Equilibrium (MDE) within each sample, as implemented in BOTTLENECK 1.2.02 [26]. At selectively neutral loci, the expected heterozygosities calculated from allele frequencies data (*He*) and from the number of alleles and sample sizes (*Heq*) are expected not to be significantly different in a population at MDE. Comparing *He *to *Heq *across loci, therefore, provides the basis for testing this hypothesis. If the population recently experienced a bottleneck, rare alleles will be rapidly lost and therefore *Heq *will decrease faster than *He*. Thus, observing a significant number of loci with *He>Heq *suggests the focal population recently experienced a bottleneck while the reverse trend (i.e., *He*<*Heq*) may suggest population expansion. Estimates of expected heterozygosity under MDE were calculated assuming a Stepwise Mutation Model (SMM) and a Two Phase Model (TPM) with 10–30% indels larger than the repeat unit. Statistical significance of the deviation from MDE was assessed for each sample across all loci by the Wilcoxon signed ranks tests and sign tests available in BOTTLENECK.

Differences in effective population size (Ne) between samples might further increase estimates of genetic differentiation between populations because differences in Ne violates assumptions of the island model of population migration, assumed to hold true when devising F-statistics [19]. Estimates of "long-term" effective population size [15] were calculated for each sample based on the expected heterozygosity at each microsatellite locus assuming a SMM using the formula Neμ = {[1/(1-He)]^2^-1}/8 [15,27], where He is the expected heterozygosity under HWE and μ is the microsatellite mutation rate.

Because the average mutation rate does not vary much even between well separated species such as pigs (7 × 10^-5^, [28]) and mice (4.5 × 10^-5^, [29]), the value of 10^-4 ^proposed by Lehmann *et al *[30] for *Anopheles gambiae *was conservatively adopted for estimating *A. moucheti *long-term Ne. Nevertheless, inferences were drawn on a relative scale, using the product of Neμ as a proxy of long-term Ne for each population, therefore alleviating any bias due to incorrect estimation of the mutation rate.

In all instances where multiple tests were conducted simultaneously, the sequential Bonferroni procedure [31] was applied to adjust the nominal significance level.

## Results

### Genetic variability within populations

A total of 355 mosquitoes of the *A. moucheti *group were analysed in this study, including 223 mosquitoes from Cameroon that were previously genotyped [12]. Of these, 64 specimens collected in Tsakalakuku (DRC) were *A. m. bervoetsi *and the rest were *A. m. moucheti*. Genotypes at 10 microsatellites were determined. All loci amplified successfully in all *A. m. moucheti *populations and were highly polymorphic, showing between 11 (AM13) and 17 (AM5 and AM15) distinct alleles. By contrast, only six loci could be amplified in the *A. m. bervoetsi *sample, among which AM13 was not polymorphic (Table 1). Consequently, this population showed the lowest allele richness (even when only five polymorphic loci were considered), and lowest expected heterozygosity (Table 1), followed by the *A. m. moucheti *population collected from the island of Bufurima (Uganda). All mainland populations of *A. m. moucheti *showed similar average allele richness (range 5.53–6.66) and expected heterozygosity (range 0.771–0.833).

**Table 1:** Genetic diversity at 10 microsatellite loci in *Anopheles moucheti *from Cameroon^a^, DRC and Uganda.

		Cameroon^a^				DRC		Uganda	
					
Locus		Simbock (2n = 118)	Olama (2n = 112)	Nyabessan (2n = 108)	Mouloundou (2n = 108)	Kenge (2n = 22)	Tsakalakuku (2n = 128)	Bufumira (2n = 114)	All (2n = 710)
AM1	*Rs*	5.99	6.30	5.35	6.92	5.87	*NA*	4.41	6.46
	He	0.805	0.823	0.805	0.854	0.853	-	0.662	0.834
	Fis	+0.139	**+0.264**	**+0.347**	**+0.338**	+0.372	-	**-0.037**	**+0.287**
AM2	*Rs*	6.06	6.87	6.0	6.67	7.75	2	4.58	6.75
	He	0.807	0.847	0.812	0.836	0.895	0.503	0.719	0.845
	Fis	+0.119	+0.186	**+0.342**	**+0.174**	+0.267	-1	**+0.321**	**+0.188**
AM5	*Rs*	8.06	7.22	8.63	7.37	5.70	3.39	5.76	8.87
	He	0.881	0.862	0.898	0.870	0.723	0.639	0.772	0.906
	Fis	**+0.150**	+0.018	+0.069	+0.168	**+0.758**	-0.027	**+0.355**	+0.21
AM6	*Rs*	7.24	6.40	7.33	7.13	3.72	*NA*	2.69	7.00
	He	0.872	0.826	0.870	0.850	0.671	-	0.482	0.847
	Fis	**+0.283**	**+0.298**	**+0.263**	**+0.202**	+0.605	-	**+0.700**	**+0.382**
AM9	*Rs*	6.54	6.58	7.80	5.85	5.38	*NA*	2.31	6.63
	He	0.762	0.800	0.875	0.785	0.745	-	0.201	0.785
	Fis	-0.026	-0.011	**+0.042**	+0.039	+0.152	-	-0.074	**+0.137**
AM10	*Rs*	4.28	4.14	5.49	4.73	5.00	1.94	4.31	4.85
	He	0.704	0.698	0.790	0.764	0.775	0.260	0.636	0.738
	Fis	-0.065	+0.028	+0.110	+0.019	+0.205	+0.107	-0.159	+0.139
AM13	*Rs*	5.93	6.12	5.49	6.16	5.37	1	4.78	5.92
	He	0.823	0.824	0.802	0.830	0.737	-	0.669	0.848
	Fis	+0.008	+0.042	+0.177	+0.077	-0.091	-	-0.042	+0.292
AM15	*Rs*	7.10	6.85	8.21	7.17	6.00	6.13	1.60	7.85
	He	0.820	0.822	0.869	0.818	0.834	0.825	0.083	0.844
	Fis	**+0.188**	+0.094	+0.049	+0.119	+0.263	**+0.193**	-0.025	**+0.264**
AM16	*Rs*	5.98	6.69	6.20	6.37	6.59	1.45	3.20	6.39
	He	0.823	0.843	0.822	0.829	0.868	0.058	0.605	0.836
	Fis	+0.051	+0.101	+0.076	+0.076	+0.203	+0.327	**+0.192**	**+0.273**
AM20	*Rs*	7.17	7.22	6.06	7.16	3.88	*NA*	3.41	6.94
	He	0.844	0.832	0.788	0.848	0.634	-	0.532	0.839
	Fis	+0.218	+0.066	**+0.203**	+0.083	+0.130	-	**+0.271**	**+0.249**

Mean across all loci	*Rs*	6.44	6.44	6.66	6.55	5.53	2.65	3.71	6.77
	He	0.814	0.818	0.833	0.830	0.771	0.228	0.534	0.689
	Fis	+0.112	+0.114	+0.166	+0.180	+0.291	-0.136	+0.175	*NC*

All: refers to populations pooled. 2n, number of chromosomes scored; *Rs*, allele richness [14]; He, expected heterozygosity under Hardy-Weinberg equilibrium [51]. Fis was calculated according to Weir & Cockerham [19] and goodness of fit to Hardy-Weinberg equilibrium was estimated by the exact test available in Genepop 3.2 [17]. Bolded values: P < 0.05 after taking into account multiple tests [31]. *NA*, No PCR product could be detected; -, irrelevant because no polymorphism was detected;*NC*, not computed. ^a ^Data adapted from [12].

Hardy-Weinberg expectations were significantly rejected (P < 0.001) for seven out of 10 loci when considering the pooled samples as belonging to a single panmictic population, with heterozygote deficits being evidenced at all loci, as expected when different gene pools are mixed. At the population level, 22 out of 65 tests did not conform to Hardy-Weinberg expectations after the multi-test analysis was taken into account. Significant deviation from HWE varied across loci in a population-dependent manner. The Uganda population from Bufumira island had the highest number of loci in departure from HWE (6 of 10) while the Kenge population had the fewest (1 of 10).

Exact tests for linkage disequilibrium within each of the seven populations resulted in three significant values out of 276 comparisons after correction by the Bonferroni procedure (two in Mouloundou (AM2-AM6, AM9-AM20) and one in Simbock (AM9-AM10)). No pair of loci appeared in linkage disequilibrium in more than one population, suggesting genetic independence between loci. When the test was performed in the pooled populations, two pairs of loci (AM2-AM16 and AM2-AM20) out of 45 possible combinations showed highly significant P values (<10^-6^).

### Genetic differentiation between populations

Table 2 shows Fst estimates for all pairwise population comparisons. Genotypic frequencies were highly significantly different among samples (G-test, P < 0.001). Low to moderate levels of genetic differentiation were measured among mainland populations of *A. m. moucheti *from Cameroon and DRC, with mean Fst estimates ranging 0.009–0.049 (P < 0.001). The island population of Bufurima (Uganda) showed higher levels of differentiation with this core group, with pairwise Fst estimates in the range 0.167–0.223 (P < 0.001). The highest levels of genetic differentiation were observed in all comparisons involving the *A. m. bervoetsi *sample collected in Tsakalakuku (DRC), with Fst estimates ranging 0.343–0.448 (P < 0.001).

**Table 2:** Pairwise Fst estimates between *A. moucheti *populations from Cameroon^a^, DRC and Uganda.

		Cameroon^a^				DRC	
			
		Simbock	Olama	Nyabessan	Mouloundou	Kenge	Tsakalakuku
Cameroon^a^	Simbock						
	Olama	0.011*					
	Nyabessan	0.014*	0.009*				
	Mouloundou	0.028*	0.023*	0.017*			
DRC	Kenge	0.049*	0.037*	0.040*	0.032*		
	Tsakalakuku	0.378*	0.372*	0.343*	0.346*	0.422*	
Uganda	Bufumira	0.167*	0.172*	0.187*	0.168*	0.223*	0.448*

*P < 0.001; DRC, Democratic Republic of Congo; ^a ^pairwise Fst estimates between Cameroon populations were already published in [12].

**Figure 2:**
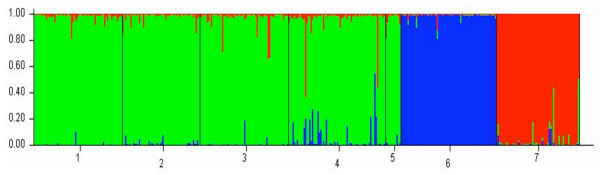
**Bayesian cluster analysis using STRUCTURE **[22]. Graphical representation of the data set for the most likely *K *(*K *= 3), where each colour corresponds to a suggested cluster and each individual is represented by a vertical bar. The numbers in the X-axis correspond to a specific sample: 1-Simbock, 2-Olama, 3-Nyabessan, 4-Mouloundou, 5-Kenge, 6-Uganda, 7-Tsakalakuku. The Y-axis represents the probability of assignment of an individual to each cluster.

In agreement with results based on Fst, the Bayesian cluster analysis showed that the most likely K value identified was K = 3. This corresponded to three distinct genetic clusters: (1) mainland *A. m. moucheti *from Cameroon and DRC, (2) *A. m. moucheti *from Bufumira island in Uganda and (3) *A. m. bervoetsi *(Figure 2).

**Figure 3:**
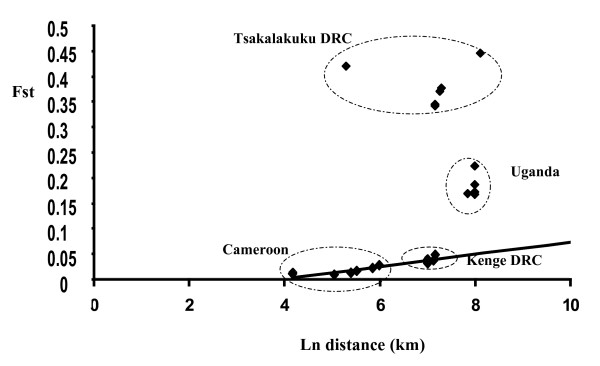
**Correlation between average Fst estimates over 10 microsatellite loci and logarithm of geographic distance between collection sites for pairwise comparisons of seven *A. moucheti *populations from Cameroon, DRC and Uganda.** The name of each sampling site refers to comparisons involving these populations.

Because isolation by distance is likely to play a major role in shaping the distribution of genetic diversity across continuous habitats [21,32], only the mainland populations of *A. m. moucheti *from Cameroon and DRC (*i.e*. excluding the samples from the island of Bufurima and the *A. m. bervoetsi *sample from Tsakalakuku and focusing on "cluster 1" described above) were used for the Mantel test. Positive and highly significant correlation (P < 0.008, Mantel test) was found between genetic (Fst) and geographic distances. Using the equation of the regression line of Fst on the logarithm of distance between sampling sites (Figure 3), the expected level of genetic differentiation between all mainland samples of *A. m. moucheti *and the *A. m. moucheti *sample from Bufurima island and *A. m. bervoetsi*, respectively were predicted under the hypothesis that geographic distance between populations was the main determinant of genetic differentiation. As can be graphically seen on Figure 3, the predicted Fst estimates were three to nine folds lower than the observed value for the Bufurima sample, and up to 8–25 folds lower than the observed value for the *A. m. bervoetsi *population. As such, distance alone contributed to less than 30% of the observed level of differentiation between the island *A. m. moucheti *sample from Bufurima and all other *A. m. moucheti *samples, while it explained less than 15% of the differentiation with the *A. m. bervoetsi *sample.

### Effective population size and demographic stability

Estimates of long-term Ne were similar for all mainland *A. m. moucheti *populations (Table 3). These were significantly lower for the Bufurima island population and the *A. m. bervoetsi *sample. Calculation of the relative ratio of Neμ compared to the *A. m. moucheti *population with the smallest effective population size (Bufumira island), showed that the estimates were five to nine folds higher for mainland populations of *A. m. moucheti*, whereas they were at least five folds lower for the *A. m. bervoetsi *sample. This demonstrates significant heterogeneity in effective population size between the different genetic clusters identified above.

**Table 3:** Long-term *Ne *estimates based on genetic diversity (expected heterozygosity) in each collection site, assuming microsatellite loci follow an SMM (see text).

Collection site		He	Ne (± SD)	Neμ	RR
Cameroon	Simbock	0.814	42,396 ± 10,934	3.488	7.7:1
	Olama	0.818	40,670 ± 10,651	3.649	8.1:1
	Nyabessan	0.833	53,046 ± 14,269	4.357	9.3:1
	Mouloundou	0.830	45,606 ± 12,092	4.20	9.3:1
DRC	Kenge	0.771	36,901 ± 24,361	2.259	5:1
	Tsakalakuku	0.228	5,230 ± 1,602	0.085	0.188:1
Uganda	Bufumira	0.534	7,983 ± 2,316	0.451	1

He, unbiased heterozygosity [15]; Ne, mean effective population size calculated across all loci (± standard deviation); μ, mutation rate; RR, relative ratio of Neμ compared to the *A. m. moucheti *population with the smallest effective population size (Bufurima island).

Estimates of genetic differentiation and effective population size however are based on the assumption of MDE. Results of the heterozygosity tests (Table 4) did not reveal any evidence for departure from MDE in any of the mainland populations of *A. m. moucheti*, nor in the *A. m. bervoetsi *population. However, a consistent trend for lower-than-expected heterozygosity (i.e., He<Heq) was evidenced for the Bufurima island population, suggesting recent demographic expansion.

**Table 4:** Estimates of P-value for the heterozygosity tests for each population of the *A. moucheti *group.

Collection site			TPM			SMM
				
			70%^a^	80%^a^	90%^a^	
Cameroon	Simbock	*He>Heq*	7	6	4	3
		*Sign test*	0.360	0.619	0.192	0.066
		*Wilcoxon test*	0.322	0.695	0.625	0.024*
	Olama	*He>Heq*	7	6	5	3
		*Sign test*	0.377	0.609	0.396	0.067
		*Wilcoxon test*	0.275	1	0.492	0.084
	Nyabessan	*He>Heq*	8	8	6	4
		*Sign test*	0.158	0.149	0.608	0.184
		*Wilcoxon test*	0.010*	0.016*	0.625	0.432
	Mouloundou	*He>Heq*	7	5	4	2
		*Sign test*	0.362	0.376	0.183	0.015*
		*Wilcoxon test*	0.557	0.846	0.275	0.019*
DRC	Kenge	*He>Heq*	7	7	7	7
		*Sign test*	0.355	0.395	0.351	0.376
		*Wilcoxon test*	0.019*	0.105	0.432	0.557
	Tsakalakuku	*He>Heq*	4	4	4	3
		*Sign test*	0.212	0.212	0.225	0.575
		*Wilcoxon test*	0.625	0.625	0.625	1
Uganda	Bufurima	*He>Heq*	2	2	1	1
		*Sign test*	0.015*	0.015*	0.002**	0.002**
		*Wilcoxon test*	0.032	0.010*	0.003**	0.002**

TPM, two-phase mutation model with ^a ^% single step mutation; SMM, stepwise mutation model.

*He>Heq*, number of loci with He>Heq (out of 10 loci tested in each sample, except the *A. m. bervoetsi *sample from Tsakalakuku where only 5 polymorphic loci were considered). * P < 0.05 and ** P < 0.01 (two tails P-values for deviation from MDE) after correction for multiple testing.

## Discussion

In this study, six *A. m. moucheti *populations from different geographic locations and one *A. m. bervoetsi *population were compared for variation in polymorphism and allele distribution at 10 microsatellite loci. Successful amplification at each microsatellite locus was obtained for all *A. m. moucheti *specimens while only six loci could be amplified in the *A. m. bervoetsi *sample, one of which did not show any polymorphism, all specimens investigated showing the same single allele at a homozygous state. This result provides further support for speciation within the *A. moucheti *group of malaria vectors in Central Africa and reflects, for the first time, genome-wide differentiation between *A. m. moucheti *and *A. m. bervoetsi*. Indeed, although the exact cytological location of the microsatellite markers is not known yet, linkage disequilibrium analysis revealed no evidence for genetic linkage between loci, suggesting they provided independent replicates for genome-wide estimation of genetic differentiation between samples. Successful amplification of microsatellite alleles was demonstrated among closely related species such as humans and great apes [33] as well as between sibling species of wasps [34] and members of anophelines species complexes [35,36]. However, the proportion of loci developed for one species that can amplify in another decreases rapidly with increasing evolutionary distance [37,38]. These results are, therefore, in straight agreement with previous studies based on morphological [7,8] and molecular data (mtDNA CytB and rDNA ITS; [10]), prompting for elevation of *A. m. bervoetsi *to full specific rank as a closely related sibling species of *A. m. moucheti*.

Linkage disequilibrium analysis further ruled out the hypothesis that the fairly high number of loci which were found out of HWE in several collections was indicative of inbreeding and/or population subdivision (within samples). If this was the case, genome-wide signatures of departure from HWE and high linkage disequilibrium between loci should be evidenced, because members of the different sub-populations would have different probabilities to carry certain combinations of alleles [30]. Such trends were not observed in the dataset, suggesting null alleles, rather than population subdivision may be responsible for the deviations observed. Null alleles are a common finding in anophelines' population genetics studies [39-41]. Because the frequencies of such null alleles might differ between sub-populations, they contribute to the overall genetic differentiation between populations. Fst estimates between populations were therefore calculated using all the information available from all loci and all samples.

Fst estimates recorded between *A. m. bervoetsi *and each of *A. m. moucheti *populations were very high and statistically significant (Fst>0.34, P < 0.001), falling in the upper range of values reported between well separated anophelines sibling species using various molecular markers [42-44]. This result, as well as results from the Bayesian analysis clearly identified *A. m. bervoetsi *as a genetically distinct entity within the *A. moucheti *group. Accordingly, it seems reasonable to consider this taxon as a full, independently evolving species within the *A. moucheti *group and, henceforth, to refer to this species as *Anopheles bervoetsi*. However, considering that *A. bervoetsi *has never been reported to occur in sympatry with *A. moucheti s.s*., nor outside of its type locality, this assertion should be validated through traditional crossing experiments, which are yet impossible to implement because members of the *A. moucheti *group have never been maintained successfully under insectary conditions. Preliminary analysis of 237 field-collected *A. bervoetsi *specimens after ELISA detected three females infected by *Plasmodium falciparum *(Antonio-Nkondjio C, Ndo C, Awono-Ambene HP and Simard F, unpublished). Although incrimination of this species as a malaria vector through dissection of its salivary gland still has to be processed, this points to a possible and previously unrecognized role of this mosquito in malaria transmission in Central Africa.

Significant isolation by distance was revealed between mainland *A. m. moucheti *populations from Cameroon and DRC, separated by distances >1,000 km (Figure 1), suggesting continuous habitat suitability for *A. moucheti *in these forested environments. Extrapolating the level of differentiation expected under the sole influence of geographical distance between mainland populations and the *A. m. moucheti *population collected on the island of Bufurima on Lake Victoria (Uganda) showed that the observed level of differentiation was three to nine folds higher than expected. Such high Fst estimates probably reflect the contribution of large water bodies separating this island population from mainland ones, acting as a barrier to gene flow by restricting opportunities for migration between populations, as was demonstrated for *A. gambiae *in this area [41] and elsewhere [36,45,46]. Moreover, significant differences in effective population sizes (Ne) were demonstrated, the island population of Bufurima showing significantly lower Ne than its mainland counterparts, and these might further increase Fst estimates [47]. Although Ne estimates based on He are criticizable because they rely on a number of assumptions including correct estimation of microsatellite mutation rate and mutation model, populations at MDE and selective neutrality of the loci, the comparison of Neμ on a relative scale allowed relieving some of these assumptions. Lower effective population size on the islands of Lake Victoria was indeed demonstrated for *A. gambiae *compared to neighbouring mainland populations [41] and the results presented here suggest the same probably applies within the *A. moucheti *group. As mentioned above however, estimates of Ne derived from He are sensitive to deviation from MDE. No significant deviation from MDE was evidenced within *A. m. moucheti *populations, although, to some extent, trends for recent population expansion were revealed for the island population of Bufurima. Colonization of Lake Victoria islands by anthropophilic malaria vectors probably followed initial human settlements on these islands in the early 1900s [41,48]. Initial founder effect might have occurred at that time but experimental studies and simulations have shown such events are only detectable for a small number of generations before a new equilibrium is reached, especially when considering molecular markers with high mutation rates such as microsatellite loci [26,49]. Inferences suggesting population expansion are generally more robust but still need to be ascertained through in-depth investigations [25].

Clearly, a more comprehensive picture of the genetic structure and distribution of genetic diversity within and among natural populations of members of the *A. moucheti *group of malaria vectors would have been obtained with the inclusion in this study of specimens of *A. m. nigeriensis*. However, as mentioned above, collections conducted in and around the type locality of this species were unsuccessful. Earlier investigations allowed collection of a few representative specimens [10] but sample sizes were far too small to allow reliable microsatellite allelic frequencies assessment. The Lagos area has recently undergone significant levels of anthropogenic environmental reshaping and urban expansion [50] and this might have led to a significant drop in *A. m. nigeriensis *populations, as was observed for *A. m. moucheti *in areas of southern Cameroon [4]. As formerly highlighted [6], the availability of PCR-based diagnostic tools and other molecular markers and their increased use in routine entomological surveys might allow more refined assessments of the diversity, geographic distribution ranges and relative epidemiological importance of the distinct anopheline species that constitute the extraordinary diverse and fluctuating malaria vector system in Africa. Such knowledge is of paramount importance for a comprehensive, efficient and sustainable implementation of vector control as a means to alleviate the malaria burden in Africa.

## Conclusion

In conclusion, this study provides strong support for considering *A. m. bervoetsi *as a full-rank, genetically independent, species within the *A. moucheti *group of malaria vectors. The species should henceforth be named *Anopheles bervoetsi*. However, its epidemiological role as a vector of human malaria parasites still deserves further investigation because roughly nothing is known to date on its biology and behaviour. Isolation by distance seems to be the major factor shaping *A. moucheti s.s*. populations' genetic structure throughout its distribution range across forested areas of Central Africa but significant geographical barriers to gene flow exist, as evidenced from reduced effective population size and high levels of genetic differentiation observed in a population collected from an island on Lake Victoria. Such genetically isolated populations in a geographically confined environment might be of considerable interest for a safe assessment of new and innovative vector control strategies aiming at population suppression and/or replacement, such as those based on the release of sterile or otherwise genetically altered mosquitoes.

## Authors' contributions

CAN was involved in the study design and implementation, conducted field sampling, microsatellite genotyping and data analysis,  prepared and drafted the manuscript. FS supervised the study conception and design, contributed to data analysis and drafted the manuscript. CN participated to field sampling in Cameroon and microsatellite genotyping. LM organized and greatly contributed to sampling in Uganda. PK was involved in molecular analysis and helped with markers selection resources. PAA and DF were involved in the conception of the study and revised the manuscript.
